# Artificial Intelligence-Based Family Health Education Public Service System

**DOI:** 10.3389/fpsyg.2022.898107

**Published:** 2022-05-11

**Authors:** Jingyi Zhao, Guifang Fu

**Affiliations:** ^1^Business School, Xi’an International University, Xi'an, China; ^2^Department of Applied Psychology, Guangdong University of Foreign Studies, Guangzhou, China

**Keywords:** artificial intelligence, family health education, public service system, internet of things technology, database system

## Abstract

Family health education is a must for every family, so that children can be taught how to protect their own health. However, in this era of artificial intelligence, many technical operations based on artificial intelligence are born, so the purpose of this study is to apply artificial intelligence technology to family health education. This paper proposes a fusion of artificial intelligence and IoT technologies. Based on the characteristics of artificial intelligence technology, it combines ZigBee technology and RFID technology in the Internet of Things technology to design an artificial intelligence-based service system. Then it designs the theme of family health education by conducting a questionnaire on students’ family education and analyzing the results of the questionnaire. And it designs database and performance analysis experiments to improve the artificial intelligence-based family health education public service system designed in this paper. Finally, a comparative experiment between the family health education public service system based on artificial intelligence and the traditional health education method will be carried out. The experimental results show that the family health education public service system based on artificial intelligence has improved by 21.74% compared with the traditional family health education method; compared with the traditional family health education method, the health education effect of the family health education public service system based on artificial intelligence has increased by 13.89%.

## Introduction

In recent years, with the substantial improvement of computer processing power, related software and platforms have also developed rapidly, and technologies such as video games and 3D simulation have also been developed at a high rate. At the same time, artificial intelligence technologies that can improve the capabilities of the entire system are also placed in an important position.

Family health education is a planned, organized, and systematic educational activity for family members. Family health education helps family members to form good behavior habits and lifestyles, reduce and eliminate risk factors affecting health, and improve the quality of life. If the family health public service based on artificial intelligence can be designed, it will play a very important role in the family health education of students. With the rapid popularization of the application of information technology in the medical field and people’s increasing attention to their own health status, the concept of health care is also changing from the original disease treatment to early prevention. The direction of self-diagnosis through personal or family health information service platform has changed from going to the hospital or clinic to be diagnosed by a doctor. People begin to pay more attention to relying on convenient health information service platform for self-preliminary diagnosis, in order to save precious time and unnecessary trouble. This paper also designs a questionnaire for students’ family education, designs a database based on the results of the questionnaire, tests its performance, and finally optimizes the artificial intelligence-based family health education public service system based on the data.

The innovation of this paper lies in the organic integration of artificial intelligence technology and Internet of Things technology. It combines artificial intelligence technology and ZigBee technology and RFID technology of Internet of Things technology to realize a public service system based on artificial intelligence and encrypts data, which ensures the security of data.

## Related Work

The fields of neuroscience and artificial intelligence (AI) have a long and intertwined history. Hassabis D believes that a better understanding of biological brains could play a crucial role in building intelligent machines. Hassabis D believes that a better understanding of biological brains could play a crucial role in building intelligent machines. He surveys the historical interaction between the fields of artificial intelligence and neuroscience and highlights current advances in artificial intelligence that are inspired by neurocomputing research in humans and other animals (Hassabis et al., 2017). His academic research in the field of artificial intelligence and neuroscience is relatively in-depth, but the application of artificial intelligence is not extensive enough. The impact of the industrial and digital (information) revolution has undoubtedly had a major impact on nearly every aspect of our society, our lives, our companies, and our jobs. By studying similar inventions of the industrial, digital, and artificial intelligence revolutions, Makridakis S claims that the latter is the goal that will bring about widespread changes that will also affect every aspect of our society and life (Makridakis, 2017). To assess the 60-min oral health education workshop for pediatric and family medicine residents in improving their knowledge, attitudes. They included the likelihood of oral health prevention practices into their current practice of healthy children’s visits as well as for the identification and referral of patients with dental trauma. Bracho P A’s pre-test and post-test designs were designed to evaluate the immediate effects of a 60-min PowerPoint oral health education workshop for pediatric and family medicine residents (Bracho et al., 2018). NB-IoT is designed to support extremely low-power and low-cost devices under extreme coverage conditions. NB-IoT operates at a very small bandwidth and will provide connectivity to a large number of low data rate devices. Beyene Y D highlights some key features introduced in NB-IoT and shows performance results from real experiments (Beyene et al., 2017). In today’s world, home security is becoming more and more necessary as the possibility of intrusions increases day by day. The design of Sisavath C is based on the design concept of “the Internet of Things is close to life and easy to use,” and builds a smart home system based on the Internet of Things. The modules included in his design include escape board module, node module, and APP module (Sisavath and Yu, 2021). Zhang X introduced the technical characteristics of NB-IoT, and he proposed a design scheme of urban lighting system based on NB-IoT. It puts more emphasis on the design of the single-lamp control system. Its hardware circuit is mainly composed of sensor module, main microprocessor, NB-IoT communication module, output module, universal user identification module, power supply module, and pulse dimming module (Zhang, 2020). The fault diagnosis of rotating machinery is of great significance to the reliability and safety of modern industrial systems. As an emerging field of industrial applications and an effective solution for fault identification, artificial intelligence (AI) technology has received increasing attention from both academia and industry. However, artificial intelligence methods face great challenges under different practical operating conditions. Liu R attempted to conduct a comprehensive review of artificial intelligence algorithms in rotating machinery fault diagnosis from both theoretical background and industrial application (Liu et al., 2018). To sum up, most of the literature on artificial intelligence and Internet of Things technology is not in-depth enough in the family health education public service system, so this paper focuses on the application of the Internet of Things technology in artificial intelligence in the family health education public service system.

## Design Method of Family Health Education Public Service System Based on Artificial Intelligence

### Artificial Intelligence

**Figure 1:**
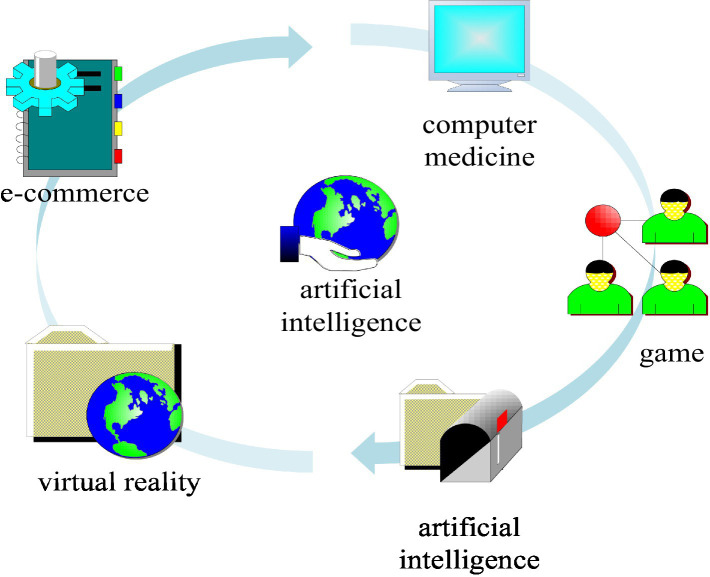
Application areas of artificial intelligence.

Artificial intelligence (AI) (Lu et al., 2017; Bertino et al., 2021; Liang et al., 2021) is an important field of computer science, a boundary topic belonging to the intersection of natural and social sciences, and it is considered one of the 3 most cutting-edge technologies of the 21st century. In the past few years, several computer systems with artificial intelligence have been built around the world to control spacecraft and underwater robots. People use programs that enable computers to perform thinking and reasoning. In this way, it is possible to master high-level human ingenuity such as environmental adaptation, automatic learning, and autonomous thought decision. With the rapid improvement of the domestic economy and the gradual implementation of the informatization strategy, the intelligence level of all walks of life in China has continued to develop, and various AI systems have been gradually established. These AI systems have penetrated into all fields of society and all aspects of people’s lives, such as e-commerce, computer medicine, mechanical engineering, games, and virtual simulation (Yang et al., 2021; Zl et al., 2021). Figure 1 shows a field related to artificial intelligence.

At the same time, in recent years, the game industry has developed rapidly around the world, and its production levels are also different. In current games, in addition to more realistic picture performance, more 3D sound effects, and more natural interaction methods, non-role players also play a more important role in the game, and the interaction between players is very important. Today, the dominant technology in game development is AI-oriented. Artificial intelligence technology is increasingly supported by developers in the virtual simulation and gaming industries.

Intelligent simulation is an important task of artificial intelligence. The way artificial intelligence is implemented is similar to the thinking process of the human brain. People obtain information about external conditions through the five senses of sight, hearing, smell, taste, and touch. Then, through the process of analysis and inference to obtain information and then make a decision. Related technologies mainly include retina and iris recognition, fingerprint and fingerprint recognition, facial recognition, automatic proof of theorems, games and logical reasoning, information detection and processing, etc.

In the daily use of mobile platforms, AI technology has matured (Glauner et al., 2017; Rim et al., 2021; Xiang et al., 2021). For example, artificial intelligence technology has contributed a lot from the lock screen of passwords and gestures to the current iris and face unlock. Mobile devices are also equipped with several input devices or sensing devices such as microphones, cameras, fingerprint readers, light sensors, gravity sensors, etc. At the same time, mobile games tend to make full use of platform functions to improve the gaming experience. For example, existing games that control a target object in a game, recognize the volume level of an incoming call, and move it up and down are simple applications of sensor-based artificial intelligence. In addition, artificial intelligence assistants such as Apple’s Siri and Microsoft’s Cortana are also common applications in daily life.

#### Definition of Artificial Intelligence

Artificial intelligence can be illustrated in two ways. On the one hand, artificial intelligence comes from the continuous progress and development of human beings, and it is the crystallization of human wisdom and civilization; on the other hand, artificial intelligence is a computer or other. It imitates the functions and actions of a part of the human electronic machine. Artificial intelligence is the study of methods that use computers to simulate the thought processes and intelligent actions of specific humans. In a word, artificial intelligence is the theory and application of computer systems, which is the development of an artificially constructed human consciousness and thinking pattern that can perform some tasks in place of humans.

At present, the domestic artificial intelligence industry structure can be divided into three parts: foundation, technology, and application support. Participants at the basic support level include data resource and computing power providers, including computing platforms and specialized hardware; participants at the technical level mainly refer to technology companies with algorithms as their core competitiveness, which provide artificial intelligence algorithm platforms or engines; the participants at the application level mainly refer to the use of artificial intelligence algorithms to develop related applications for specific problems. At this stage, the leading enterprises in the domestic intelligent industry mainly study core algorithms, starting from the basic layer; small and medium-sized startups start from the technical and application support, mainly conduct research on speech, visual recognition, etc., and then bring artificial intelligence into products and services in various fields such as security, finance, and education.

With the attention of the country and the needs of the times, the number of artificial intelligence enterprises in China has increased rapidly in recent years, and the scale of financing has expanded rapidly. The fields it involves have expanded from unit to diversified development, which also means that artificial intelligence will gradually become an auxiliary force for various industries in the future, and will even become an indispensable key factor for China’s economic development in the future.

#### Artificial Intelligence Research Content

The study of artificial intelligence is very technical and specialized. Each branch is deep, scattered, and covers a wide area. The research contents of artificial intelligence are mainly knowledge representation and automatic inference, retrieval methods and knowledge processing, machine learning and knowledge acquisition, computer vision, and natural language understanding, automatic programming, and intelligent robots.

#### Characteristics of Artificial Intelligence

The artificial intelligence industry mainly has the following characteristics:

First, knowledge-intensive industries have greater demand for talents and higher costs. Artificial intelligence is an industry based on deep learning computer algorithms and data collection, which requires a large number of high-end technical talents. In recent years, the popularity of artificial intelligence has also caused the shortage of talents in professional fields, thereby increasing the cost of talents. At present, there is still a large gap in the supply of talents in the artificial intelligence industry.

Second, the artificial intelligence industry is a structural system with rich participants. It provides services for intelligent core technology by the basic support composed of data supply and computing power. It applies smart products to various scenarios such as medical care, finance, home furnishing, and education through commercial construction.

Third, artificial intelligence is an evolution and supplement to traditional industries. The rise of artificial intelligence has not completely replaced the status of traditional industries, but it is an improvement of traditional industry products. Artificial intelligence is applied to traditional industries, supports traditional industries through cash technology, and increases cooperative development and commercial implementation of traditional industries.

Fourth, each segment of the artificial intelligence industry presents specialization, and there are differences in the overall degree of correlation. At present, the artificial intelligence industry has a broad layout, and the technical research of the latter field has a high degree of professionalism. However, there are limitations in the development of various fields, artificial intelligence technologies in various fields cannot be perfectly integrated, and there are certain differences in technical correlations.

Fifth, artificial intelligence is in the stage of exploration and development. Although artificial intelligence has been in development for a long time, it is still in its early stages. Certain achievements have been made in perception technology, but there is still a breakthrough in the development of cognitive technology; the development bottleneck of semantic recognition is relatively large; object recognition and scene recognition of computer vision are still under research. The development of artificial intelligence in the future is still full of unknowns and expectations, and the artificial intelligence industry will create a different future.

#### The Main Problems Currently Faced by Artificial Intelligence Technology

(1) Running the framework puzzle. Its thinking ability is designed and taught by humans, but human beings have not yet found an algorithm that can make the machine better reach the level of human brain thinking, that is, the operating framework of artificial intelligence. Only by mastering the operating mechanism of the brain can scientists break through the existing bottleneck of artificial intelligence.

(2) Citizens are concerned about the development of artificial intelligence. Since artificial intelligence was invented in human society, the social impact of artificial intelligence has received extensive attention in human society. In society, some people also expressed concern about the emergence and development of artificial intelligence and intelligent robots and worried that artificial intelligence and intelligent robots would one day threaten the survival and development of human beings. There are other views in society. Although artificial intelligence has made great progress after 60 years of development, the overall level of artificial intelligence will still be difficult to surpass human intelligence in the near future, and it will not be enough to threaten the survival of human beings. They argue that the impact of artificial intelligence on human society must be taken very seriously, the concerns of citizens should be ignored, and no time should be spent on research and development of countermeasures to ensure human safety.

#### Major Key Areas of Artificial Intelligence Technology

The research of artificial intelligence as a whole has just begun, and it is still far from our goal, but artificial intelligence will have a big breakthrough in some aspects.

(1) Machine learning (Thrall et al., 2018; Zhu et al., 2020) is an interdisciplinary technology in many fields including probability theory, statistics, approximation theory, convex analysis, algorithmic complexity theory, and other fields of technology. It continues to improve performance by reorganizing existing knowledge structures, which is the core of artificial intelligence technology and the basic method for computers to have intelligence. Some existing reinforcement learning algorithms are not very practical enough, but human beings always believe that artificial intelligence will go further on this road under the joint research of scientists.

(2) Natural language processing (Cath et al., 2017; Jain and Mahanti, 2020) is an important direction in the field of computer science and artificial intelligence. Scientists and scientific research institutions in many countries are devoted to natural language processing of computers. Through the efforts of researchers, this field has resulted in many noteworthy theories and applications such as Apple’s SIRI. Natural language processing is not the general study of natural language, but the development of computer systems, especially software systems, that can effectively realize natural language communication. Once artificial intelligence realizes natural language, it will further develop in depth and will significantly improve the “intelligence” of artificial intelligence.

### IoT Technology

The concept of IoT was originally proposed in 1999 (Bui et al., 2017). That is to say, the Internet of Things is through radio frequency identification technology equipment, infrared sensors, global positioning system GPS, laser scanners, and other information transmission equipment. It is the internet of things that is connected to any item *via* a specific network protocol. It is mainly used to realize information exchange and communication network for intelligent identification, positioning, tracking, monitoring and management.

Based on the technology of the Internet of Things, medical equipment can tend to develop intelligently and innovatively. Compared with traditional medical products, intelligent medical equipment shows intelligent characteristics such as real-time perception, information recognition, data analysis, and action decision-making. If medical equipment no longer relies on traditional methods for diagnosis and treatment, modern intelligent products combined with Internet of Things technology will provide better services for human health.

**Figure 2:**
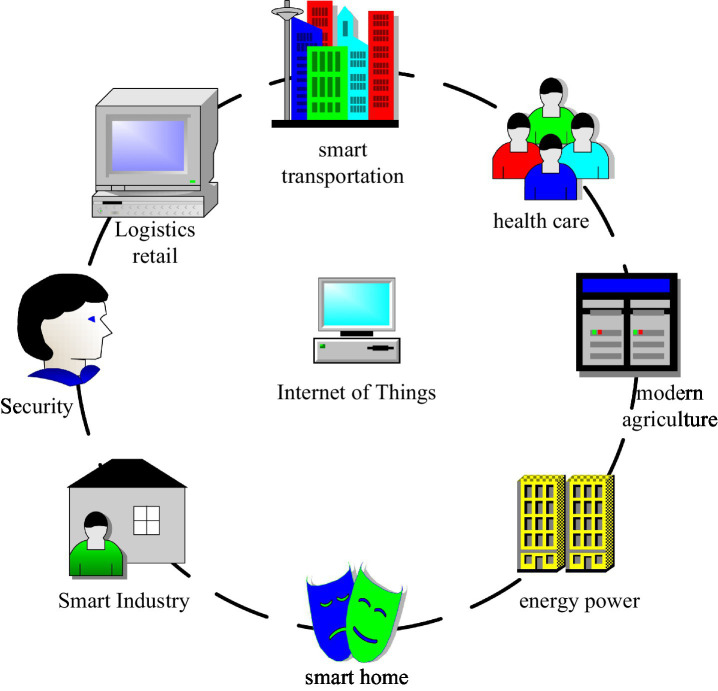
IoT application areas.

As shown in Figure 2, the Internet of Things also has huge development and application achievements in the fields of security, logistics and retail, health care, and modern agriculture. It uses advanced technological means to improve the overall level of all walks of life. Especially in recent years, the health care Internet of Things has provided a good and effective industrial platform for the whole people to pay attention to health issues, making the medical system more perfect. Not only the treatment of diseases, it also provides high-quality health services.

#### ZigBee Technology

The origin of ZigBee (Nasr et al., 2017; Wu et al., 2017) is the honeybee, and its action principle is similar to that of the honeybee, when a bee sees a bee, it needs to call other bees and use its own body movement to convey location information, which the same process as ZigBee technology sends information. After the terminal node collects the signal, it informs the coordinator or router of its own node address and hands it to other terminal nodes. ZigBee has 3 working frequency bands, and ZigBee can work on these 3 frequency bands. The global frequency band is 2.4GHz, the European frequency band is 868 MHz, and the US frequency band is 915 MHz. The transmission distance of ZigBee is 10–75 meters, and the biggest feature of ZigBee is that it consumes very little power. As a result, the system brings great advantages: the power exchange period becomes longer, the stability of the system is improved, and manpower and financial resources are saved, so ZigBee has been widely used.

**Figure 3:**
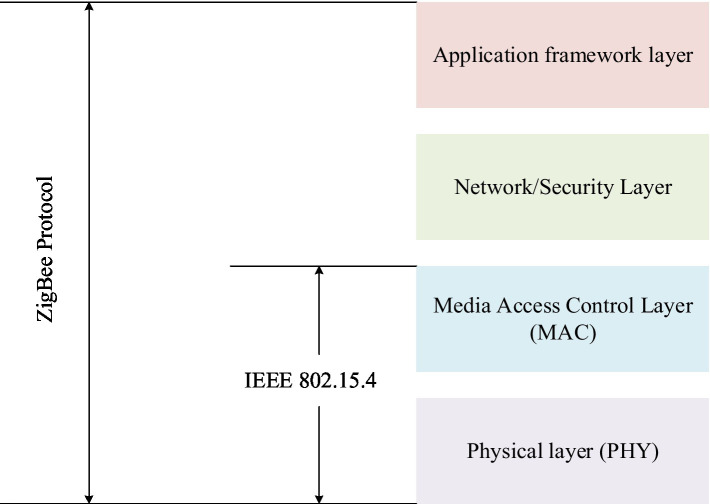
ZigBee architecture diagram.

ZigBee technology is a wireless communication technology with high reliability and low-power consumption. The architecture of ZigBee technology consists of four layers: physical layer (PHY) (Baum, 2017), media access control layer (MAC) (Burton et al., 2017), network/security layer (Price and Flach, 2017), and application framework working layer. The distribution between the layers is shown in Figure 3.

##### Physical Layer

The physical layer is the interface between the MAC layer and the physical wireless channel, and it provides interface services through RF firmware and RF hardware. The physical layer consists of two parts, the data service access point and the management entity access point, which, respectively, provide data services and management services for the physical layer. The data service of the physical layer supports the transfer of MAC protocol data units between two peer MAC layer entities, and the management service of the physical layer allows the transfer of management commands between the MME and the PLME.

##### Media Intervention Control Layer

The MAC sublayer handles access to all physical layer wireless communications. The functions of the MAC sublayer are: synchronization with beacons generated by the network coordinator, responsible for establishing and disconnecting LAN links, using the operator multi-access mechanism, avoiding conflicts between different devices and network nodes, reliable communication links between peer entities, etc.

##### Network/Security Layer

The function of the network layer is to ensure the normal operation of the media intervention layer and provide interface services to the application layer. The network layer includes two functional entities, the data service entity and the management service entity. The data service entity mainly provides the data transmission service of the access point, and the management service entity mainly provides the network management service of the access point.

##### Application Frame Layer

The application layer consists of three parts: ASP sublayer, ZDO device object, and application object. The ASP sublayer is composed of ASP data entity and ASP management entity.

**Figure 4:**
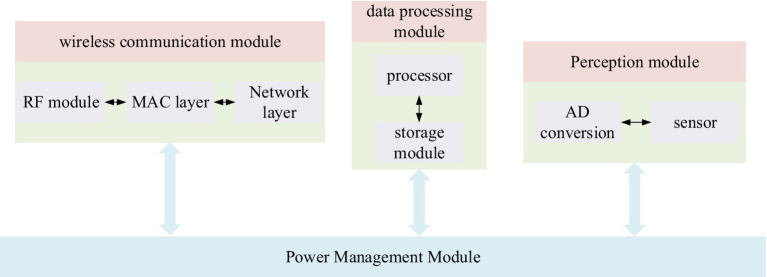
ZigBee node hardware model diagram.

ZigBee node is mainly composed of: perception module, data processing module, wireless communication module, and power supply module. Figure 4 shows the hardware model diagram of the ZigBee node.

As shown in the figure above, the perception module consists of a sensor module and an AD conversion module. The sensor module completes the collection of environmental information and transmits it as an analog quantity. After the analog quantity is obtained, the AD conversion module converts it into a digital quantity that can be recognized by the system, and finally, the data processing module completes the data processing operation.

#### RFID Technology

**Figure 5:**
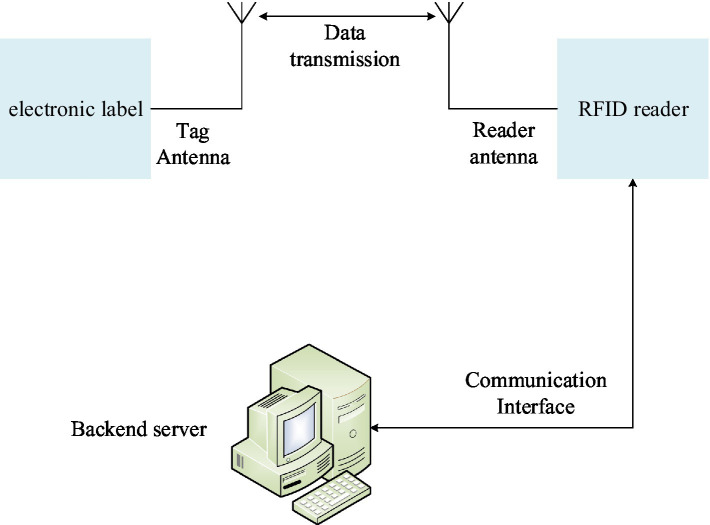
Structure and composition of RFID system.

The basic operating principle of the RFID system: After the RFID card receives the radio frequency signal from the reader, the antenna coil obtains the induced current, and at the same time supplies power to the chip through the booster circuit to obtain the data information of the induced current. It is controlled by logic, the circuit processes the information, and the resulting data is sent back to the reader through the antenna through the logic circuit. Therefore, the antenna plays an important role in realizing the data communication between the RFID card and the card reader. On the one hand, the passive RFID card chip must gain enough energy in the magnetic field generated by the reader antenna through the antenna to start the circuit. On the other hand, the antenna determines the communication channel and communication method with the RFID card. The RFID system is mainly composed of three parts: RFID tag, reader, and background server. The system structure is shown in Figure 5.

The electronic tags in the system store the data information required by users, and the number of each RFID tag is unique. The reader needs to write the information to the tag while acquiring the information of the RFID tag, which has the advantages of large data storage space and strong data processing capability. The background server usually saves and processes the information of the RFID tag identified by the reader and the information sent by the reader.

### Key Generation Method

The relay-assisted key generation method is divided into three stages: channel detection, relay-assisted, and key negotiation. *X* and *R* need to send sounding signals, respectively, for channel estimation, occupying 3 time slots, the relay station occupies 1 time slot, and the key negotiation needs to occupy 1 time slot. The key generation process must be completed within a coherence time, so the coherence time T can be equally divided into 5 time slots $S_{1},S_{2},S_{3},S_{4},S_{5}$, namely


(1) $$S_{1}=S_{2}=S_{3}=S_{4}=S_{5}=S/5$$

**Figure 6:**
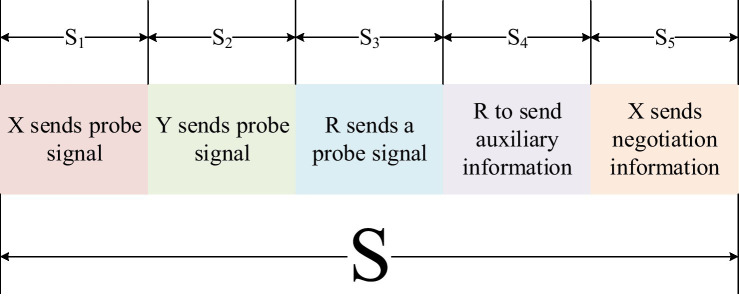
Time slot allocation diagram in a coherence time S.

The time slot allocation is shown in Figure 6.

In the time slot, *X* sends a known probe signal $a_{X}$, and *Y* receives the signal at this time


(2) $$b_{0.Y}=h_{0}a_{X}+k_{0,Y}$$

*R* received the signal


(3) $$b_{1.R}=h_{1}a_{X}+k_{1,R}$$

Among them, $k_{0,Y}$ and $k_{1,R}$ are the received noises of *Y* and *R* in the current time slot, respectively. *Y* estimates *h0* and gets


(4) $${\tilde{h}}_{0,Y}=h_{0}+\frac{a_{X}^{S}}{a_{X}{}^{2}}k_{0,Y}$$

Where ${\left(*\right)}^{S}$ represents the transpose of the vector or matrix. *R* estimates *h1* and gets:


(5) $${\tilde{h}}_{1,R}=h_{1}+\frac{a_{X}^{S}}{a_{X}{}^{2}}k_{1,R}$$

In the time slot $S_{2}$, *Y* sends a known probe signal, and *X* receives the signal at this time


(6) $$b_{0.Y}=h_{0}a_{Y}+k_{0,Y}$$

*R* received the signal


(7) $$b_{2.R}=h_{2}a_{Y}+k_{2,R}$$

Among them, $k_{0,X}$ and $k_{2,R}$ are the received noises of X and R in the current time slot, respectively. X according $b_{0,X}$ to the estimated h2, get


(8) $${\tilde{h}}_{0,X}=h_{0}+\frac{a_{Y}^{S}}{a_{Y}{}^{2}}k_{0,X}$$

*R* estimates *h2* from $b_{2,R}$, and gets


(9) $${\tilde{h}}_{2,R}=h_{2}+\frac{a_{Y}^{S}}{a_{Y}{}^{2}}k_{2,R}$$

In time slot $S_{3}$, *R* sends the known probe signal $a_{R}$, and *X* receives the signal at this time


(10) $$b_{1.X}=h_{1}a_{R}+k_{1,X}$$

*Y* receives the signal


(11) $$b_{2.Y}=h_{2}a_{R}+k_{2,Y}$$

Among them, $k_{1,X}$ and $k_{2,Y}$ are the received noises of *X* and *B* in the current time slot, respectively. *X* estimates *h1* from $b_{1,X}$, and gets


(12) $${\tilde{h}}_{1,X}=h_{1}+\frac{a_{R}^{S}}{a_{R}{}^{2}}k_{1,X}$$

*Y* estimates *h2* according to $b_{2,Y}$, and we get


(13) $${\tilde{h}}_{2,Y}=h_{2}+\frac{a_{R}^{S}}{a_{R}{}^{2}}k_{2,Y}$$

It is easy to get Gaussian random variable ${\tilde{h}}_{0,X},{\tilde{h}}_{0,Y},{\tilde{h}}_{1,X},{\tilde{h}}_{1,R},{\tilde{h}}_{2,Y},{\tilde{h}}_{2,R}$ whose mean is 0. Assuming that the powers of *X*, *Y*, and *R* sending $a_{X},a_{Y},a_{R}$ are all *P*, the variance of ${\tilde{h}}_{0,X}$ is


(14) $$\sigma^{2}=\sigma_{0}^{2}+\frac{\sigma_{k}^{2}}{PS/5}$$

Then


(15) $${\tilde{h}}_{0,X}\sim N\left(0,\sigma_{0}^{2}+\frac{\sigma_{k}^{2}}{PS/5}\right)$$

The same can be obtained


(16) $${\tilde{h}}_{0,Y}\sim N\left(0,\sigma_{0}^{2}+\frac{\sigma_{k}^{2}}{PS/5}\right)$$

(17) $${\tilde{h}}_{1,X}\sim N\left(0,\sigma_{1}^{2}+\frac{\sigma_{k}^{2}}{PS/5}\right)$$

(18) $${\tilde{h}}_{1,R}\sim N\left(0,\sigma_{1}^{2}+\frac{\sigma_{k}^{2}}{PS/5}\right)$$

(19) $${\tilde{h}}_{2,Y}\sim N\left(0,\sigma_{1}^{2}+\frac{\sigma_{k}^{2}}{PS/5}\right)$$

(20) $${\tilde{h}}_{2,R}\sim N\left(0,\sigma_{1}^{2}+\frac{\sigma_{k}^{2}}{PS/5}\right)$$

However, the common information between *X* and *Y* at this time is only the estimated value of the direct channel *h0*.

## Test Experiment of Family Health Education Public Service System Based on Artificial Intelligence

### The Main Body of Family Education Guidance Work

**Figure 7:**
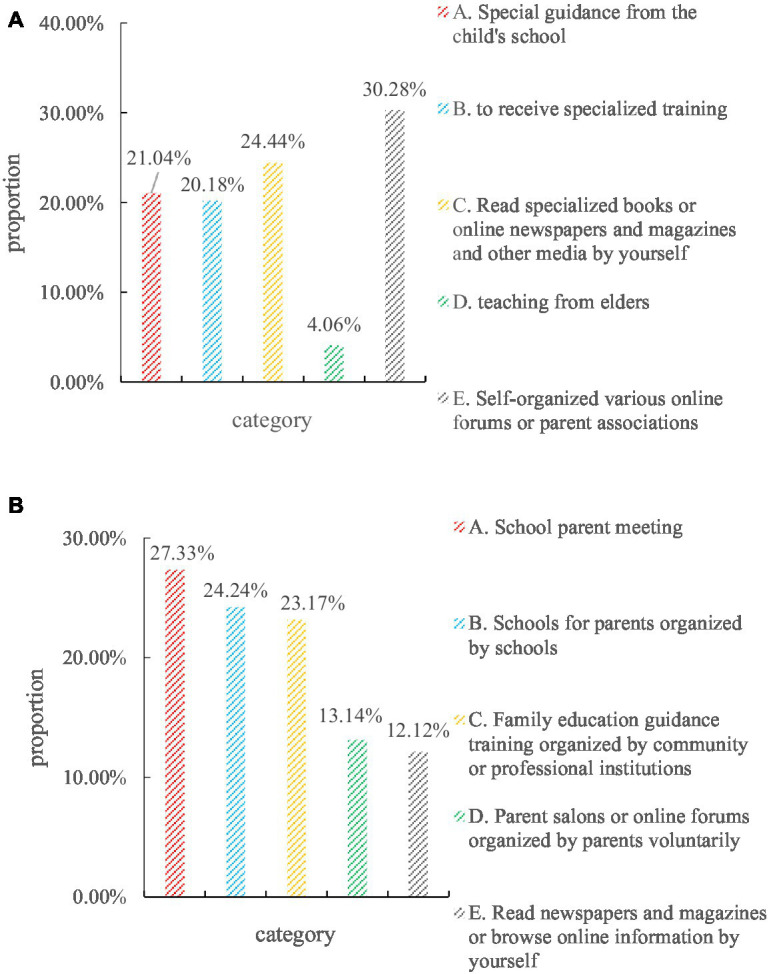
Questionnaire survey results. **(A)** Parents consider the most important source of family education-related knowledge ideally. **(B)** Parents are most willing to accept or consider the most effective form of home education guidance.

In this survey, the questionnaires were distributed to parents of school students. According to the situation of parents of different age levels and children’s education level, it is divided into three grades of primary school, junior high school, and high school, and 100 questionnaires are distributed in each grade. The research questionnaire collected 300 questionnaires from 10 primary and secondary schools in 5 districts, and 292 were recovered, with a recovery rate of 97.33%. The results are shown in Figure 7.

Whether it is the current actual situation or the parents’ own wishes, the main responsibility of family education guidance is the school where the children are located. For example, in the question of “Your main source of knowledge about family education,” “special guidance from your child’s school” accounted for 27.33%; in the question of “Do you think the most important source of knowledge about family education should be,” 23.17% chose “special guidance from the child’s school”; 24.24% of the respondents chose “school parent meeting” in the question of “what do you most like or think is the most effective form of family education guidance.” Regardless of the value of these statistical results, they are the options with the highest proportion.

**Figure 8:**
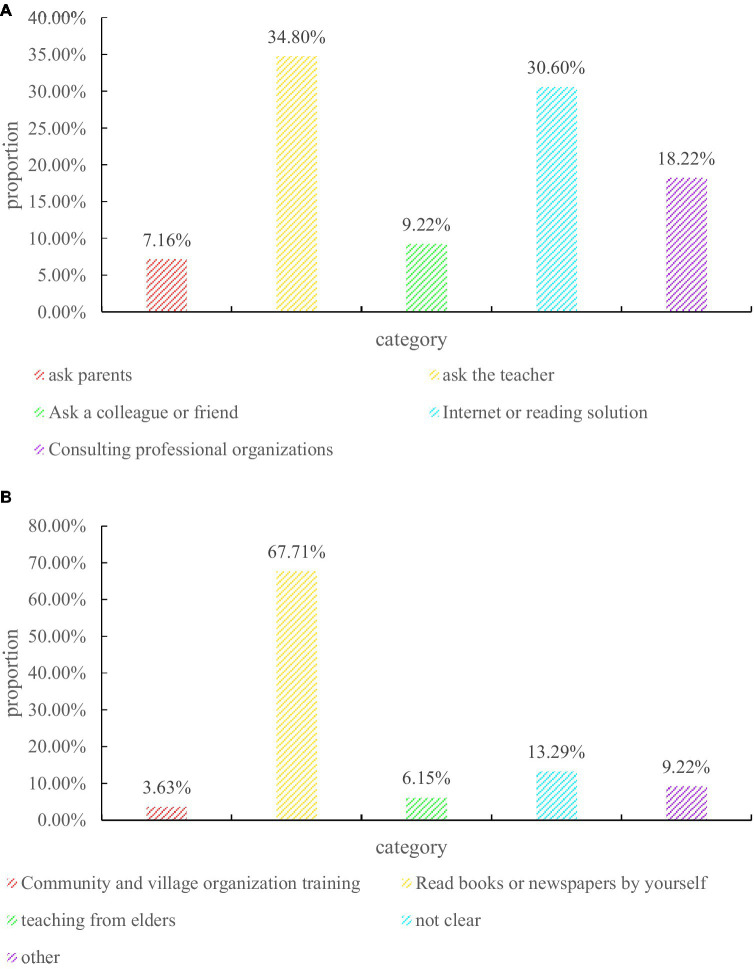
Questionnaire for Student Education Questions. **(A)** How to deal with family education problems. **(B)** Primary source of educational knowledge.

In the questionnaire survey of parents that schools should be the main provider of family education public services, the results are shown in Figure 8.

Aiming at the question of who is the main provider of family education public services, the questionnaire designed a situation of “when you encounter family education problems, how do you usually deal with it” to obtain the attitude of the respondents. The five options set are: A, ask their parents for advice; B, take the initiative to ask the head teacher or other teachers of the school for advice; C, ask colleagues or friends; D, search for solutions on the Internet or read books; F, consult professional institutions. Finally, the distribution of each option selected by the respondents is shown in the figure. We found that choosing option B to take the initiative to consult school head teachers or other teachers accounted for the largest proportion, accounting for almost 40%, followed by consulting their parents and professional institutions. This also shows that in the eyes of most parents and students, they have more trust in the home education guidance provided by the school.

As one of the participants in children’s family education, the family education provided by parents is a very important part of the children’s education, and the source and method of parents’ obtaining family education-related knowledge affects the actual effect of family education. However, at this stage, what are the ways for parents to obtain knowledge about family education? What are the main ways? These are extremely important issues in the study of family education. Accordingly, this question is also set in the questionnaire, and some common ways to obtain family education knowledge are listed for the respondents to choose. The statistical results are shown in the figure, we found that nearly 70% of the respondents said they obtained knowledge of family education by reading special books or through online newspapers and magazines. This shows that they are trying to actively participate in the process of family education, and most of them acquire the relevant knowledge of family education through self-study, and it also shows that their awareness of self-education is increasing.

### Database Module Design

Using SQLite database. General mobile platforms such as iOS and Android use SQLite database, which belongs to a light database.

The data information includes the following: basic information of family members, user history, cloud server IP and port, etc. The design of the data table is described below.

The family member basic information table AllUserInfo contains the family member’s ID number, name, gender, date of birth, nickname, height, weight, login password, and the user’s avatar, and the family member is its unique identifier. The user’s avatar is several different avatars set in the application, and different avatars have different numbers, so the designed data type is Char type.

The detailed design of each field is shown in Table 1.

**Table 1:** Basic information of family members.

Column name	Type of data	Describe	Primary key
User ID	Int	User ID number	Yes
Name	Varchar (100)	Name	No
Sex	Char (10)	Gender	No
Birth	Datetime (8)	Date of birth	No
Nick	Varchar (100)	Nickname	No
Height	Real	Height	No
Weight	Real	Weight	No
Password	Varchar (100)	Password	No
Portrait	Char (10)	Set avatar	Primary key

The cloud server IP port table Domain contains the IP address and port of the cloud network server. The detailed design of each field is shown in Table 2.

**Table 2:** Port domain name table.

	Server ip	Port
Type of data	Varchar (50)	Varchar (50)
Describe	Server ip address	Server port
Primary key	No	No

The user history table UserData contains the user’s ID number, sensor device type, sensor ID number, weight value, body temperature value, blood pressure value, and measurement time. The detailed design of each field is shown in Table 3.

**Table 3:** User history table.

Column name	Type of data	Describe	Primary key
Userid	Int	User id number	Yes
Sensortype	Varchar (100)	Sensor device type	No
Sensorid	Char (10)	Sensor id number	Yes
Value0	Real	Weight	No
Value l	Real	Body temperature	No
Value2	Rea1	Height	No
Value3	Real	Blood pressure	No
Time stamp	Text	Measure time	No

The set-top box number table STBIndex contains the number of the set-top box. When the mobile phone remotely accesses the cloud server, it needs to obtain the corresponding user data through the index number of the set-top box. The detailed design of each field is shown in Table 4.

**Table 4:** Set-top box number table.

Column name	Type of data	Describe	Primary key
Stbindex 1	Int	Box number 1	Yes
Stbindex 2	Int	Box number 2	No
Stbindex 3	Int	Box number 3	No

The authority division table is shown in Table 5.

**Table 5:** User permission table.

Numbering	User	Group/role	Permission
1	Guest	Tourists	Measurement, diagnosis
2	Family member	General user	Measure, save data, diagnose, history, unbind devices
3	ADMIN	Administrator	Add user, delete user, unbind device, change server IP, port

## Artificial Intelligence-Based Family Health Education Public Service System

### Heart Rate Evaluation Results of IoT Devices Based on Artificial Intelligence

**Figure 9:**
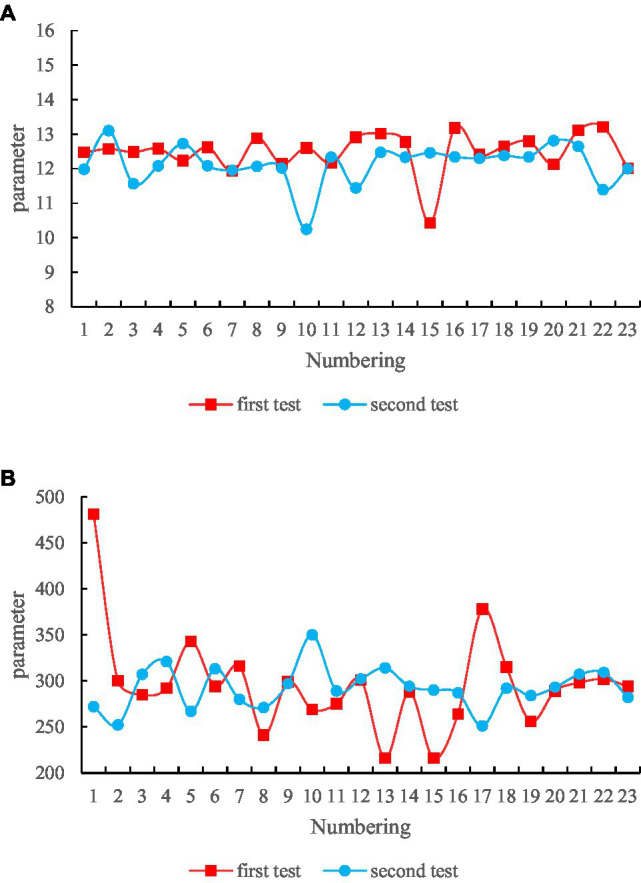
Compression result (20% DCT filter window). **(A)** CR parameter results. **(B)** Running time comparison.

In this paper, 48 groups of ECG signals in the MIT-BIH arrhythmia database are used to test the compression algorithm, and the first 100 s (36,000 points) data of the first lead is selected for each signal for testing. The algorithm is run on HPZ228 computer (64-bit Windows 7 operating system, processor: Intel Core i7-4,790 CPU@3.6 GHz, installed memory: 8.00 GB). The comparison between our method and other compression methods is shown in Figure 9.

It can be seen from the figure that using adaptive run-length coding for data compression has a low compression ratio and serious distortion; empirical mode decomposition or wavelet transform compression method can obtain higher compression ratio, but the distortion of reconstructed signal is serious; the method of Huffman coding with all transform domain data of DCT obtains the lowest distortion rate, but its compression rate is low; using a 20% filter window to intercept the DCT data of the signal, the highest compression ratio can be obtained, and the distortion degree is within the acceptable range. In this paper, on the basis of the filtering window intercepting the DCT signal for data compression, the process of using Huffman coding to further compress the signal is abandoned. This is because, in the process of variable-length coding based on the statistical probability of the data, the Huffman coding value and coding table of different heartbeat data are different, which makes the home gateway need to transmit data at the same time. The encoding table for each encoding, and also need to transmit the encoding table of each encoding. On the one hand, it increases the data processing time and consumes more system resources such as storage space of the home gateway. On the other hand, it brings inconvenience to the decoding of the data.

### Test Score Analysis of Artificial Intelligence-Based Family Health Education Service System

**Figure 10:**
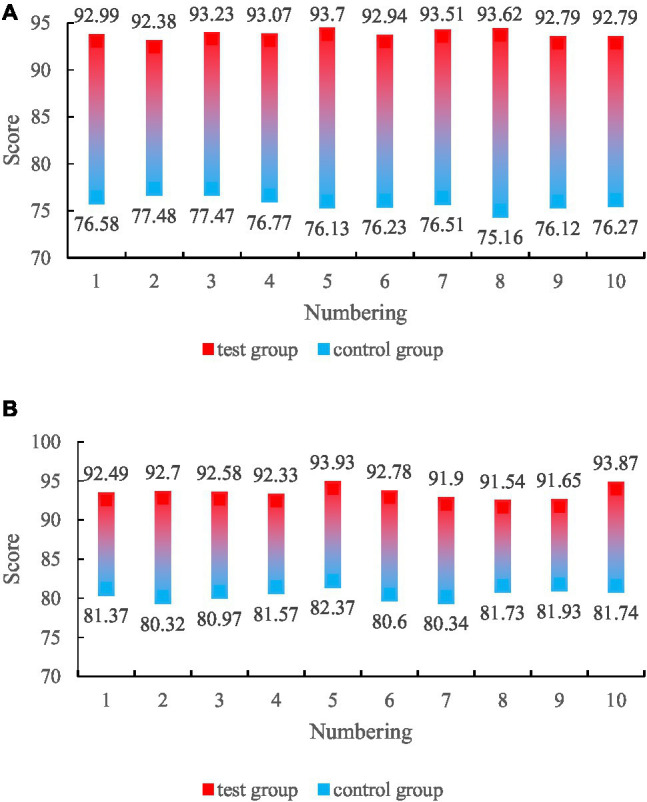
Results scores of health education methods in the two groups. **(A)** Health education method score. **(B)** Health education effect score.

The data collected through the experiment has finally optimized the artificial intelligence-based family health education public service system designed in this paper. One group used the artificial intelligence-based family health education public service network system designed in this paper for health education, and the other group adopted the traditional health education method. There were 10 students in the two groups. The test results are shown in Figure 10.

As can be seen from the figure, the health education method score of the artificial intelligence-based family health education public service system reached 93.10 points, while the score of the traditional family health education method was only 76.47 points. It can be seen that the score of the family health education public service system based on artificial intelligence has increased by 21.74% compared with the traditional family health education method; the health education effect score of the health education method of the family health education public service system based on artificial intelligence reached 92.58 points; however, the health education effect score of the traditional family health education method is only 81.29 points, it can be seen that the health education effect score of the family health education public service system based on artificial intelligence has increased by 13.89% compared with the traditional family health education method. It can be concluded from the experiments that the educational methods and educational effects of the artificial intelligence-based family health education public service system are very popular among students, which is very helpful to family health education.

## Conclusion

This paper mainly studies the design of the family health education public service system based on artificial intelligence. Therefore, this paper focuses on the characteristics and technologies of artificial intelligence, and then combines the ZigBee technology and RFID technology in the Internet of Things technology to design the family health education public service system based on artificial intelligence, and then the data security is ensured by encrypting the data key, so that the system in this paper can run safely. This paper designs the main body analysis experiment and database design of family education guidance work to conduct a questionnaire on students’ family education, and then analyzes the results of the questionnaire to design the focus of this paper. Then this paper designs an analysis of the heart rate evaluation results of IoT devices based on artificial intelligence, analyzes the performance of the device, and analyzes and discusses the data obtained from the experiment, and finally optimize the family health education public service system based on artificial intelligence designed in this paper. In the final comparative experiment, the experimental results show that: Compared with the traditional health education method, the artificial intelligence-based family health education public service system is very popular with students for its educational methods and educational effects.

## Data Availability Statement

The original contributions presented in the study are included in the article/supplementary material, further inquiries can be directed to the corresponding author.

## Ethics Statement

Ethical approval for this study and written informed consent from the participants of the study were not required in accordance with local legislation and national guidelines.

## Author Contributions

JZ: work concept or design and draft paper. GF: data collection and make important revisions to the paper. All authors contributed to the article and approved the submitted version.

## Conflict of Interest

The authors declare that the research was conducted in the absence of any commercial or financial relationships that could be construed as a potential conflict of interest.

## Publisher’s Note

All claims expressed in this article are solely those of the authors and do not necessarily represent those of their affiliated organizations, or those of the publisher, the editors and the reviewers. Any product that may be evaluated in this article, or claim that may be made by its manufacturer, is not guaranteed or endorsed by the publisher.
